# The impacts of the Covid-19 pandemic, policy responses and macroeconomic fundamentals on market risks across sectors in Vietnam

**DOI:** 10.1371/journal.pone.0272631

**Published:** 2022-08-23

**Authors:** Hung Quang Bui, Thao Tran, Hung Le-Phuc Nguyen, Duc Hong Vo

**Affiliations:** 1 University of Economics Ho Chi Minh City, Ho Chi Minh City, Vietnam; 2 International School of Business, University of Economics Ho Chi Minh City, Ho Chi Minh City, Vietnam; 3 Research Centre in Business, Economics & Resources, Ho Chi Minh City Open University, Ho Chi Minh City, Vietnam; Bucharest University of Economic Studies: Academia de Studii Economice din Bucuresti, ROMANIA

## Abstract

Vietnam has undergone four waves of the Covid-19 pandemic in 2020 and 2021, which have posed significant market risks to various sectors. Understanding the market risk of Vietnamese sectors and its changes is important for policy implementation to support the economy after the pandemic. This study measures the sectoral market risks and examines the effects of the pandemic, policy responses and macroeconomic fundamentals on the market risks across sectors in Vietnam. We employ the Value-at-Risk (VaR) and Conditional Value-at-Risk (CVaR) techniques to measure the market risks for 24 sectors from 2012 to 2021. The market risk levels across Vietnamese sectors have changed significantly in response to the pandemic. *Oil and Gas* and *Services* sectors show the largest potential loss during the two Covid-19 waves in 2020. *The Securities* sector is the riskiest sector during the last two Covid-19 waves in 2021. Our results indicate that the new Covid-19 cases reported by the Government increase the market risk levels across Vietnamese sectors. On the other hand, enhancing containment and health policy and reducing economic policy uncertainty result in lower market risk across sectors. We also find that macroeconomic fundamentals such as the exchange rate and interest rate significantly affect the market risks across sectors in Vietnam.

## 1. Introduction

During the 1970s, companies were exposed to significant risks that emerged from the disappearance of fixed-currency parities, volatility of commodity prices, and natural disasters. These sources of risk, in the form of unprecedented events, revolutionize the concept of risk management in the financial sector [1]. Over nearly half a century, risk management has evolved with unparalleled events, typically the Global Financial Crisis (GFC) of 2007–2008 and the current Covid-19 pandemic. Such events are often associated with the change in market risk, thus making market risk management one of the top priorities for companies and investors worldwide.

In acknowledging the necessity of effective market risk management, various modern risk management models have been introduced and adopted to address market risk proactively. Typical models in previous studies include the Maximum Loss [2], Expected Shortfall [3], Value-at-Risk [4–6], and Conditional Value-at-Risk [7, 8]. Generally, the Value-at-Risk (VaR) and the Conditional Value-at-Risk (CVaR) are the most widely used risk measurements to observe risk movements in specified markets, such as the stock markets [3, 9–11], commodity market [12], as well as foreign exchange and cryptocurrency market [6]. Previous studies have focused exclusively on the market risk of industries in Australia [13], Europe [14], and ASEAN members [8] for the pre-and post-GFC period.

Vietnam has achieved stable economic growth in the past three decades until the emergence of the pandemic in 2020. The national economy grew steadily in the first two quarters of 2021. Still, it declined significantly in the third quarter of the year when the Covid-19 pandemic severely hit Ho Chi Minh City, the country’s largest economic and financial centre. The services sector is a main contributor to the Vietnamese economy. As such, the downturn in services-related business activities reduced the overall growth of the services sector and the entire economy. The services suffering the largest declines include accommodation and food, transportation and warehousing, and wholesale and retail. Fortunately, growth in the agriculture and industry sectors (agriculture, forestry, fisheries, processing and manufacturing, electricity production and distribution) and other services-related sectors (finance, banking and insurance, information and communication) have saved Vietnam’s economy from recession [15]. Such an unbalanced growth across sectors in Vietnam poses significant risks to entrepreneurs and investors, especially during the unpredictable Covid-19 pandemic, which is still ongoing in 2022. Understanding the market risk, its changes, and the effects of policy responses to the current pandemic and macroeconomic fundamentals are important for policy implementation to support the national economy. This important consideration forms the basis for our study to be conducted.

Recently, the Covid-19 emergence has further highlighted the importance of understanding the sectoral market risk, especially for the Vietnamese sectors, which have accumulated limited experience in risk management from significant events. Our literature review indicates that only one study was recently conducted to examine the market risk of the Vietnamese sectors and further examined the relationship between the market risk and the Covid-19 pandemic in Vietnam [16]. Other studies have focused on the connectedness among different markets with the exposure to market risk from the Covid-19 pandemic [17–21]. Meanwhile, previous studies have confirmed significant effects of the Covid-19 pandemic [22–25], policy responses [23–30] and macroeconomic fundamentals [31–34] on stock market return and volatility.

Recent studies have mainly focused on the effects of the Covid-19 pandemic and policy responses on stock market return and volatility. The effects of macroeconomic fundamentals have largely been ignored in previous studies. Our study is different from previous analyses. We examine the impacts of the pandemic, relevant policy responses and macroeconomic fundamentals on the market risk of 24 Vietnamese sectors from 2012 to 2021. This study uses VaR and CVaR methods to measure the market risk in Vietnam. Our study makes the following contributions to the existing literature. First, we estimate the market risk at the sectoral level in Vietnam, which has largely been ignored in previous studies. Second, we investigate the changes in market risk in the Vietnamese sectors in the last decade, including 2020 and 2021, with the emergence of the Covid-19 pandemic. Third, we then examine the effects of the pandemic, policy responses to the pandemic and macroeconomic fundamentals on market risk across 24 sectors in Vietnam.

The paper is structured as follows. Following this introduction, section 2 discusses and synthesizes related literature. Next, data and methodology are presented and discussed in section 3. Section 4 reports and discusses the empirical results, followed by the concluding remarks and implications in section 5 of the paper.

## 2. Literature review

The Value-at-Risk (VaR) is the maximum loss at a certain confidence level in a given holding period. The VaR appears to be the most widely adopted risk measurement technique. Risk managers and investors use the VaR to implement long-term capital management plans and estimate expected losses [4]. However, the most significant limitation of the VaR is that the method does not consider the scenarios where the VaR estimates are exceeded [35, 36]. In other words, VaR may underestimate the risk level because the method ignores all returns worse than the estimated VaR at the given confidence level. Rockafellar et al. [7] introduce the Conditional Value-at-Risk (CVaR), which can capture the expected losses exceeding the estimated VaR at the same confidence level. CVaR provides an average expected loss rather than a wide range offered by VaR that is difficult to account for. Therefore, VaR and CVaR are widely used separately or together to measure and observe risk movements in different areas.

Kourouma et al. [3] examine the risk of the French stock market index, S&P 500, wheat, and crude oil during the GFC using VaR and Expected Shortfall. Terinte [11] employs VaR to examine the risk of five stocks in the Romanian financial market from 2011 to 2015. Powell et al. [12] developed ECVaR, a modified CVaR metric, to investigate the tail risk of several commodities relative to the S&P Goldman Sachs Commodity Index (GSCI) for different economic cycles. Uyar et al. [6] employ VaR to analyze the risk of Bitcoin and conventional currencies.

In addition, Rout et al. [10] employ VaR and CVaR to measure the national stock market risks across 20 countries (G20) for the 1998–2020 period. The research period is divided into four regimes covering (i) the Asian financial crisis, (ii) the internet bubble bursting, (iii) the GFC, and (iv) the Covid-19 pandemic. Findings from their analysis indicate a significantly high level of market risk across these 20 countries during the GFC and the Covid-19 pandemic. Among the four regimes, risks during the Covid-19 pandemic highlight the most significant magnitude. Li et al. [9] employ VaR and CVaR to investigate whether Covid-19 new cases and deaths magnify the equity market risk in China, the UK, and the US. They conclude that an increase in Covid-19 new cases or deaths is associated with a significant increase in the market risk across the three countries.

From the industry perspective, Allen et al. [13] utilised VaR and CVaR to examine risk across 25 Australian industries for 15 years based on equity price movements. In another study, Allen et al. [14] compare the market risk of the European industries before and during the GFC using various VaR and CVaR techniques. Their results show that the riskiest sectors before the GFC are different from those during the GFC. Vo et al. [8] apply CVaR to examine risk, returns, and portfolio optimization at the industry level for four ASEAN nations, during the 2007–2016 period covering the GFC. Their study reveals that Vietnam and Malaysia witness the shift in rankings of the best sectoral performance from the post-GFC to the normal period.

The market risk across sectors appears to change significantly with the extreme events. Ho et al. [16] estimate the market risks across ten Vietnamese sectors before and during the Covid-19 pandemic (2012–2020). Their analysis uses VaR and CVaR to measure the market risk. Their study is then extended to examine the effect of the Covid-19 pandemic on the risk of these Vietnamese sectors. The results show that risks across sectors in Vietnam have surged after the Covid-19 outbreak. However, the market risk decreases during the entire lockdown period. No analysis has been conducted concerning the relationship between the pandemic, policy response, macroeconomic fundamentals and the market risk across sectors in Vietnam.

Meanwhile, previous studies have found significant effects of the Covid-19 pandemic [22–25], policy responses [23–30], and macroeconomic fundamentals [31–34] on stock market returns and volatility. Specifically, increased Covid-19 new cases are associated with lower stock market returns [22–24], while the effect on stock market volatility is mixed [25]. Regarding the policy responses, an increase in the containment and health index is associated with positive stock market returns [23, 24, 27] and mitigates market volatility [25]. Meanwhile, heightened economic policy uncertainty is related to negative market returns [26, 30] and strengthens volatility in the short run [28, 29]. LIBOR is found to have significant explanatory power for stock returns [34]. Gold returns predetermine stock returns in the short run during the Covid-19 pandemic [32]. Exchange rate fluctuation has a significant causal relationship with stock return for pre-and post-inflation targeting periods [33] and Covid-19 policy responses [31].

Market risk will also be affected by the pandemic, policy responses and macroeconomic fundamentals due to market volatility and changes in market return. However, the current strands of literature only focus extensively on examining the connectedness among different markets with the exposure to market risk from the Covid-19 pandemic, such as energy markets [17, 18], infrastructure markets [20], stock markets [21], and economic indicators [19]. Key findings from these studies suggest that the Covid-19 pandemic intensified the connectedness among the markets.

In addition, Akyildirim et al. [17] indicate a strong connectedness between energy markets worldwide with high uncertainty and low economic sentiment. Hernandez et al. [18] find that oil price volatility during the Covid-19 period exerts a causal effect on the connectedness dynamics across US stock sectors. Susantono et al. [20] recommend using the infrastructure assets to hedge against the USD index and USD-denominated assets due to their persistent negative connection. Uddin et al. [21] present a significant predictive power from the world financial index to the regional market indices. Regarding predictability, Qureshi [19] finds that the Covid-19 pandemic significantly affects the long-term predictive economic variables, which can portend the future economic state.

Our literature review indicates that only Ho et al. [16] examine the effects of the Covid-19 pandemic on the market risk across sectors in Vietnam. Their analysis, albeit exciting and recent, has primarily ignored relevant and important factors, such as policy responses and macroeconomic fundamentals, which potentially affect the market risks across sectors. Our literature review confirms that our analysis examining the effects of the Covid-19 pandemic, policy responses and macroeconomic fundamentals across 24 sectors in Vietnam during the ten years is warranted.

## 3 Data and research methodology

### 3.1. Data

We obtain the daily sector index of 24 sectors in Vietnam to estimate the market risk. Data are collected from Ho Chi Minh City Stock Exchange (HOSE) and Hanoi Stock Exchange (HNX) from 03 January 2012 to 15 September 2021. These 24 sectors include *Aquaculture*, *Aviation*, *Banking*, *Building Materials*, *Business*, *Construction*, *Construction Investment*, *Development Investment*, *Education*, *Energy*, *Fertilizer*s, *Food*, *Mineral*s, *Oil and Gas*, *Pharmaceutical*s, *Plastic*, *Real Estate*, *Rubber*, *Securities*, *Services*, *Steel*, *Technology*, *Trade*, and *Transportation*.

We use VaR and CVaR to measure the market risk across these 24 sectors. We then examine the effect of the pandemic, policy responses and macroeconomic fundamentals on the market risk of these 24 sectors during the ten years. We use the daily new confirmed Covid-19 cases to proxy the pandemic, consistent with previous studies [22–24]. The Containment and Health Index (CHI) and Economic Policy Uncertainty (EPU) index are used to proxy the policy responses to the Covid-19 pandemic [37, 38]. These choices follow recent studies indicating the significant effects of CHI [23–25, 27] and EPU [26, 28–30] on stock market returns and volatility during the Covid-19 pandemic.

We finally use the exchange rate (USD/VND), interest rate (LIBOR), and gold prices to capture the effects of macroeconomic fundamentals on the market risks. These macro-level measures have significant relationships with the stock market returns [31–34]. All variables are summarized in Table 1.

**Table 1:** Selected variables, descriptions, and sources.

Variable	Description	Source
**Dependent variable**		
Market risk	Market risk across 24 Vietnam sectors	HOSE and HNX
**Independent variables**		
Covid-19 pandemic	New confirmed Covid-19 cases	Our World in Data
Policy responses	Containment and Health Index (CHI)	Our World in Data
	Economic policy uncertainty (EPU)–Equity Market Volatility: Infectious Disease Tracker	FRED | St. Louis Fed (stlouisfed.org)
Macroeconomic fundamentals	Exchange rate (USD/VND)	Thomson Reuters—Refinitiv
	LIBOR—Swiss 3-month LIBOR Middle rate (SNB)	
	Gold price–Gold Bullion LBM $/t oz	

### 3.2. Methodology

The market returns of each sector are calculated using the logarithmic returns of the daily closing prices of the sector indices. Consequently, we adopt VaR and CVaR as our risk measurement methods. For VaR calculation, the following three standard estimation techniques are generally used in the existing literature, including the Variance-Covariance method, Monte Carlo simulation, and historical simulation [39]. The variance-covariance (or the parametric) approach is used in our study. This parametric approach is a widely used method that assumes returns follow a normal distribution. We then calculate the mean and standard deviation of all sectors’ returns before estimating the market risk, as demonstrated by McNeil et al. [40].

$$VaR_{\alpha }=\mu +\sigma .f_{\left(\alpha \right)}^{-1}$$

*where*: *μ* is the mean of all returns from a sector; *σ* is the standard deviation; $f_{\left(\alpha \right)}^{-1}$ is the inverse of the normal distribution of the returns, and 1-α represents the confidence level.

We also employ the parametric approach for CVaR estimation:

$$CVaR_{\alpha }=E(r\left|r\geq VaR_{\alpha }\right.)$$

where: *r denotes the returns of a particular sector*

We use VaR and CVaR to estimate the yearly and monthly market risk across 24 sectors in Vietnam. Our yearly VaR and CVaR capture the annual market risk of each sector from 2012 to 2021. Meanwhile, the monthly VaR captures the monthly market risk of each sector between January 2020 and September 2021. We also rank each sector’s market risk based on the yearly VaR and CVaR, together with monthly VaR.

We use the fixed-effects estimation to examine the effect of the Covid-19 pandemic, policy responses and macroeconomic fundamentals on the market risk across 24 sectors in Vietnam. The monthly market risk estimated from the VaR technique is used as a dependent variable. We then convert the daily closing indices of all the independent variables, including new confirmed Covid-19 cases, Containment and Health Index, Economic Policy Uncertainty, Gold prices, Exchange rate, and LIBOR, into monthly closing indices. We also use the logarithmic transformation for the monthly value of these variables except for LIBOR. A panel of 24 sectors and 21 months (from January 2020 to September 2021) is used.

$$Market\;risk_{it}=\alpha_{0}+\beta_{1}Cases_{t}+\beta_{2}CHI_{t}+\beta_{3}EPU_{t}+\beta_{4}Exchange_{t}+\beta_{5}LIBOR_{t}+\beta_{6}Gold_{t}+\varepsilon_{it}$$

*where*: *i* and *t* stand for the sector and the month. *Cases* denote the percentage change in monthly new Covid-19 cases. *CHI* is the percentage change in the Containment and Health Index. *EPU* represents the percentage change in the Economic Policy Uncertainty Index. *Exchange* is the percentage change in the exchange rate (USD/VND). *LIBOR* is the value of the LIBOR interest rate. *Gold* is the percentage change in the gold price, and *ε* represents the error term.

## 4. Empirical results

### 4.1. Market risk across sectors in Vietnam

Table 2 presents the descriptive statistics of the daily returns on 24 sector indices in Vietnam from 03 January 2012 to 15 September 2021. We obtain 2,420 daily observations to calculate the market risks yearly and monthly across 24 sectors in Vietnam. *Aviation* has the highest average daily return of 0.146 per cent. *The Mineral*s sector shows the lowest average daily return of 0.002 per cent. When risks are considered using the standard deviation of the daily returns, *Pharmaceutical* is the least risky sector. In contrast, *Aviation* is the riskiest sector among the 24 sectors in Vietnam.

**Table 2:** Descriptive statistics of the daily return of 24 sectors in Vietnam.

No.	Sector	N	Mean (%)	SD (%)	Min (%)	Max (%)
1	Aquaculture	2,420	0.094	1.529	-7.147	5.622
2	Aviation	2,420	0.146	2.700	-14.14	16.58
3	Banking	2,420	0.053	1.536	-7.302	5.712
4	Building Materials	2,420	0.077	1.471	-8.729	5.096
5	Business	2,420	0.083	1.147	-9.397	7.076
6	Construction	2,420	0.081	1.739	-8.090	7.353
7	Construction Investment	2,420	0.049	1.430	-7.169	5.058
8	Development Investment	2,420	0.063	1.991	-11.66	11.37
9	Education	2,420	0.061	1.610	-7.869	9.422
10	Energy	2,420	0.084	1.178	-6.551	5.031
11	Fertilizer	2,420	0.054	1.435	-7.890	5.786
12	Food	2,420	0.053	1.197	-7.291	4.580
13	Mineral	2,420	0.002	1.937	-13.21	11.36
14	Oil & Gas	2,420	0.040	1.920	-8.686	6.423
15	Pharmaceutical	2,420	0.072	1.091	-6.240	5.643
16	Plastic	2,420	0.070	1.284	-6.358	5.272
17	Real Estate	2,420	0.049	1.426	-7.079	5.557
18	Rubber	2,420	0.012	1.458	-7.013	4.909
19	Securities	2,420	0.085	1.880	-7.941	6.712
20	Services	2,420	0.065	1.910	-9.548	9.081
21	Steel	2,420	0.091	1.788	-10.66	7.598
22	Technology	2,420	0.090	1.299	-7.252	5.902
23	Trade	2,420	0.080	1.452	-7.049	5.766
24	Transportation	2,420	0.063	1.179	-7.139	5.228

Table 3 presents the yearly market risks based on VaR (Panel A) and CVaR (Panel B) across 24 sectors in Vietnam. Generally, the market risks calculated by VaR and CVaR at a 95 per cent confidence level have an average of 2.42 per cent and 3.03 per cent, respectively, over the 2012–2021 period. *Aviation* appears to be the riskiest sector, with a VaR of 3.83 per cent and CVaR of 4.79 per cent. Meanwhile, *Pharmaceutical* has the lowest risk level among all sectors with a VaR of 1.70 per cent and CVaR of 2.13 per cent.

**Table 3:** The yearly market risks of 24 sectors in Vietnam, 2012–2021, using VaR (panel A) and CVaR (panel B).

Year	Average	Aqua	Aviation	Banking	Build Mat	Business	Construct	Cons Inv	Dev Inv	Edu	Energy	Fertilizer	Food	Mineral	Oil & Gas	Pharma	Plastic	Real Estate	Rubber	Securities	Services	Steel	Tech	Trade	Transport
**Panel A: VaR at 95 per cent confidence level**																									
Average	2.42%	2.40%	3.83%	2.41%	2.29%	1.76%	2.65%	2.28%	2.89%	2.52%	1.81%	2.22%	1.88%	3.04%	3.06%	1.70%	2.01%	2.26%	2.35%	2.89%	2.97%	2.79%	2.01%	2.26%	1.83%
2021	2.58%	2.40%	2.39%	2.99%	2.33%	2.65%	1.92%	2.54%	0.94%	2.52%	1.90%	2.85%	2.02%	2.88%	3.49%	1.81%	1.90%	2.67%	2.80%	4.31%	3.25%	3.57%	2.47%	2.88%	2.52%
2020	2.39%	2.42%	3.43%	2.85%	2.28%	1.72%	1.96%	2.14%	1.02%	2.55%	1.69%	2.45%	2.30%	2.10%	3.43%	1.68%	1.98%	2.63%	2.13%	2.93%	3.70%	2.98%	2.21%	3.28%	1.47%
2019	1.76%	1.94%	1.73%	1.42%	1.81%	1.01%	1.52%	1.97%	2.43%	2.66%	1.41%	1.72%	1.22%	2.68%	1.96%	1.09%	1.47%	1.62%	1.67%	1.57%	2.51%	2.33%	1.48%	1.66%	1.26%
2018	2.92%	3.04%	3.21%	3.65%	3.12%	2.21%	3.43%	2.30%	3.82%	2.89%	2.09%	2.31%	1.94%	4.50%	4.44%	2.03%	2.50%	2.56%	2.66%	3.44%	2.86%	3.39%	2.42%	3.27%	1.96%
2017	1.93%	2.74%	2.12%	1.64%	1.58%	1.17%	2.57%	1.89%	3.36%	2.10%	1.82%	1.93%	1.24%	2.78%	1.94%	1.58%	1.65%	1.38%	2.28%	1.64%	2.44%	1.87%	1.47%	1.91%	1.15%
2016	2.50%	2.28%	3.66%	2.03%	1.93%	1.60%	2.63%	1.59%	2.52%	3.98%	1.37%	1.26%	1.99%	4.91%	3.21%	1.78%	1.83%	1.85%	3.55%	2.01%	4.90%	4.08%	1.39%	2.01%	1.55%
2015	2.25%	2.29%	3.18%	2.71%	2.00%	1.37%	1.92%	2.09%	3.87%	2.53%	1.27%	1.69%	1.84%	2.71%	3.36%	1.61%	1.98%	1.87%	1.84%	2.57%	3.34%	2.43%	1.77%	1.87%	1.77%
2014	2.63%	2.27%	4.19%	1.94%	2.79%	1.92%	3.33%	2.82%	5.44%	1.94%	1.93%	2.64%	1.84%	3.08%	3.27%	1.73%	2.09%	2.59%	2.16%	3.91%	1.83%	2.53%	2.69%	2.06%	2.24%
2013	2.40%	2.52%	6.40%	2.18%	2.19%	1.50%	3.37%	2.39%	2.64%	1.55%	2.22%	2.22%	2.15%	2.81%	2.40%	1.65%	2.04%	2.45%	1.90%	2.58%	2.63%	2.01%	1.96%	1.79%	1.95%
2012	2.87%	2.08%	7.98%	2.69%	2.91%	2.42%	3.89%	3.05%	2.91%	2.44%	2.43%	3.15%	2.29%	1.96%	3.08%	2.05%	2.63%	2.98%	2.53%	3.94%	2.26%	2.72%	2.25%	1.84%	2.45%
**Panel B: CVaR at 95 per cent confidence level**																									
Average	3.03%	3.00%	4.79%	3.01%	2.87%	2.20%	3.32%	2.84%	3.61%	3.14%	2.27%	2.78%	2.35%	3.78%	3.81%	2.13%	2.51%	2.82%	2.92%	3.61%	3.71%	3.49%	2.52%	2.82%	2.29%
2021	3.26%	3.04%	3.00%	3.74%	2.94%	3.36%	2.43%	3.18%	1.18%	3.16%	2.37%	3.64%	2.52%	3.67%	4.36%	2.31%	2.39%	3.33%	3.47%	5.46%	4.07%	4.52%	3.16%	3.63%	3.20%
2020	2.99%	3.07%	4.28%	3.56%	2.86%	2.17%	2.46%	2.70%	1.28%	3.15%	2.12%	3.10%	2.88%	2.65%	4.27%	2.11%	2.49%	3.27%	2.67%	3.72%	4.58%	3.77%	2.77%	4.08%	1.85%
2019	2.19%	2.41%	2.16%	1.79%	2.25%	1.26%	1.88%	2.46%	3.06%	3.31%	1.76%	2.10%	1.51%	3.32%	2.44%	1.36%	1.82%	2.02%	2.09%	1.93%	3.12%	2.89%	1.87%	2.08%	1.56%
2018	3.61%	3.81%	3.97%	4.53%	3.84%	2.75%	4.20%	2.84%	4.70%	3.57%	2.60%	2.87%	2.41%	5.57%	5.49%	2.50%	3.06%	3.19%	3.32%	4.26%	3.54%	4.20%	3.01%	4.05%	2.42%
2017	2.42%	3.39%	2.69%	2.08%	1.99%	1.45%	3.23%	2.35%	4.21%	2.61%	2.27%	2.40%	1.58%	3.48%	2.45%	2.00%	2.05%	1.76%	2.84%	2.09%	3.06%	2.35%	1.85%	2.41%	1.44%
2016	3.11%	2.83%	4.58%	2.52%	2.42%	1.98%	3.36%	1.98%	3.15%	4.96%	1.71%	1.55%	2.50%	6.10%	4.02%	2.24%	2.31%	2.30%	4.32%	2.48%	6.10%	5.08%	1.74%	2.53%	1.92%
2015	2.79%	2.84%	3.99%	3.40%	2.51%	1.73%	2.41%	2.61%	4.77%	3.17%	1.59%	2.10%	2.31%	3.30%	4.12%	1.98%	2.49%	2.31%	2.27%	3.19%	4.12%	3.01%	2.21%	2.33%	2.20%
2014	3.30%	2.88%	5.30%	2.42%	3.55%	2.41%	4.16%	3.52%	6.83%	2.43%	2.43%	3.26%	2.29%	3.83%	4.07%	2.15%	2.60%	3.24%	2.70%	4.89%	2.27%	3.15%	3.36%	2.59%	2.80%
2013	3.00%	3.13%	8.02%	2.71%	2.74%	1.91%	4.20%	2.97%	3.25%	1.97%	2.81%	2.78%	2.67%	3.45%	3.04%	2.09%	2.59%	3.05%	2.38%	3.21%	3.36%	2.53%	2.45%	2.24%	2.48%
2012	3.59%	2.61%	9.93%	3.35%	3.61%	3.02%	4.84%	3.78%	3.68%	3.08%	3.06%	3.96%	2.87%	2.42%	3.84%	2.57%	3.29%	3.70%	3.17%	4.90%	2.85%	3.41%	2.78%	2.29%	3.04%

Note: **Aqua**–Aquaculture; **Build Mat**–Building Materials; **Construct**–Construction; **Cons Inv**–Construction Investment; **Dev Inv**–Development Investment; **Edu**–Education; **Tech**–Technology; **Pharma**–Pharmaceutical; **Transport**–Transportation.

Given Vietnam’s Covid-19 outbreak in February 2020, we consider the change in market risk level across 24 Vietnamese sectors during this pandemic. Table 3 displays that most sectors witness an abrupt increase in market risk from 2019 to 2020, except for *Development Investment*, *Education*, and *Mineral*s. *Services* sector shows the most potential loss in 2020, with a VaR of 3.70 per cent and CVaR of 4.58 per cent. Meanwhile, *Development Investment* bears the lowest risk level with a VaR of 1.02 per cent and CVaR of 1.28 per cent within the same period. Also, the sector experiencing the most significant change in risk level during 2019–2020 is *Aviation*, with an increase in VaR by 1.70 per cent and CVaR by 2.12 per cent.

We then rank the market risk of 24 sectors by year to further compare the market risk level among sectors from 2012 to 2021 in Table 4. The rankings range from 1 to 24, corresponding to the highest to the lowest risk level using VaR (in panel A) and CVaR (in panel B). These market risk rankings indicate that many sectors are significantly volatile over the 2012–2021 period. Only a few sectors could maintain the risk ranking stability, such as *Pharmaceutical*.

**Table 4:** Ranking the yearly market risk of 24 sectors from 2012 to 2021 using VaR (Panel A) and CVaR (Panel B).

Year	Aqua	Aviation	Banking	Build Mat	Business	Construct	Cons Inv	Dev Inv	Edu	Energy	Fertilizer	Food	Mineral	Oil & Gas	Pharma	Plastic	Real Estate	Rubber	Securities	Services	Steel	Tech	Trade	Transport
**Panel A: Risk ranking by VaR at 95 per cent confidence level (1 indicates the riskiest sector, and 24 indicates the least risky sector)**																								
2021	16	17	5	18	11	20	12	24	13	22	8	19	7	3	23	21	10	9	1	4	2	15	6	14
2020	11	3	7	13	20	19	15	24	9	21	10	12	17	2	22	18	8	16	6	1	5	14	4	23
2019	8	10	19	9	24	16	6	4	2	20	11	22	1	7	23	18	14	12	15	3	5	17	13	21
2018	11	9	4	10	20	6	19	3	12	21	18	24	1	2	22	16	15	14	5	13	7	17	8	23
2017	3	7	16	19	23	4	12	1	8	14	10	22	2	9	18	15	21	6	17	5	13	20	11	24
2016	10	5	11	15	19	8	20	9	4	23	24	14	1	7	18	17	16	6	13	2	3	22	12	21
2015	10	4	5	12	23	14	11	1	8	24	21	18	6	2	22	13	16	17	7	3	9	20	15	19
2014	13	2	18	8	21	4	7	1	19	20	10	22	6	5	24	16	11	15	3	23	12	9	17	14
2013	7	1	14	13	24	2	10	4	23	11	12	15	3	9	22	16	8	20	6	5	17	18	21	19
2012	21	1	11	8	17	3	6	9	15	16	4	18	23	5	22	12	7	13	2	19	10	20	24	14
**Panel B: Risk ranking by CVaR at 95 per cent confidence level (1 indicates the riskiest sector, and 24 indicates the least risky sector)**																								
2021	16	17	5	18	10	20	13	24	15	22	7	19	6	3	23	21	11	9	1	4	2	14	8	12
2020	11	2	7	13	20	19	15	24	9	21	10	12	17	3	22	18	8	16	6	1	5	14	4	23
2019	8	10	19	9	24	16	6	4	2	20	11	22	1	7	23	18	14	12	15	3	5	17	13	21
2018	11	9	4	10	20	7	19	3	12	21	18	24	1	2	22	16	15	14	5	13	6	17	8	23
2017	3	7	16	19	23	4	13	1	8	14	11	22	2	9	18	17	21	6	15	5	12	20	10	24
2016	10	5	12	15	20	8	19	9	4	23	24	13	1	7	18	16	17	6	14	2	3	22	11	21
2015	10	4	5	12	23	14	11	1	8	24	21	16	6	3	22	13	17	18	7	2	9	19	15	20
2014	13	2	20	7	21	4	8	1	19	18	10	22	6	5	24	16	11	15	3	23	12	9	17	14
2013	7	1	14	13	24	2	10	5	23	11	12	15	3	9	22	16	8	20	6	4	17	19	21	18
2012	21	1	11	9	17	3	6	8	14	15	4	18	23	5	22	12	7	13	2	19	10	20	24	16

Note: **Aqua**–Aquaculture; **Build Mat**–Building Materials; **Construct**–Construction; **Cons Inv**–Construction Investment; **Dev Inv**–Development Investment; **Edu**–Education; **Tech**–Technology; **Pharma**–Pharmaceutical; **Transport**–Transportation.

*Pharmaceutical* appears to experience the lowest variation in risk rankings during the ten years. The sector’s risk rankings consistently range from 18 to 24, indicating the lowest market risk level. Further, the pharmaceutical sector maintains a low level of risk. Interestingly, *Development Investment* and *Minerals* are often the riskiest sectors in the pre-Covid-19 period from 2014 to 2019. However, this pattern has changed significantly since the pandemic emerged in 2020. Specifically, these two sectors were ranked 24th and 17th out of 24 in 2020. However, before the Covid-19 outbreak, these two sectors were ranked 4th and 1st in 2019.

The emergence of Covid-19 appears to change the market risk levels across sectors and their risk rankings in Vietnam. Therefore, we measure the monthly market risk to examine such changes during the pandemic. Tables 5 and 6 provide the monthly market risk and the ranking among 24 sectors in Vietnam. Market risk is measured using the VaR technique.

**Table 5:** Monthly market risks of 24 sectors in Vietnam from 2020 to 2021 using the VaR technique.

The market risk using VaR at a 95 per cent confidence level																									
Year	Month	Aqua	Aviation	Banking	Build Mat	Business	Construct	Cons Inv	Dev Inv	Edu	Energy	Fertilizer	Food	Mineral	Oil & Gas	Pharma	Plastic	Real Estate	Rubber	Securities	Services	Steel	Tech	Trade	Transport
2021	Sep	1.55%	2.04%	1.45%	2.33%	2.15%	1.78%	2.68%	1.00%	3.13%	1.88%	2.46%	1.02%	2.06%	2.17%	2.35%	1.14%	1.13%	1.52%	1.63%	1.19%	2.59%	1.31%	2.02%	2.58%
	Aug	2.40%	0.97%	2.46%	2.15%	2.16%	1.57%	1.83%	0.45%	1.12%	1.28%	2.48%	1.70%	1.96%	2.97%	1.68%	1.43%	2.37%	1.93%	3.14%	2.11%	2.60%	1.62%	2.41%	3.07%
	July	3.29%	2.78%	4.63%	2.17%	3.22%	1.67%	3.35%	1.12%	1.89%	1.53%	4.18%	1.72%	2.18%	4.63%	1.94%	2.59%	3.53%	3.63%	6.88%	3.42%	5.52%	3.03%	4.80%	3.08%
	Jun	2.79%	1.36%	2.80%	1.96%	2.03%	1.51%	1.48%	0.62%	2.96%	1.11%	1.90%	0.92%	1.65%	3.03%	1.32%	1.57%	1.51%	1.63%	4.81%	1.81%	3.63%	1.56%	1.51%	1.58%
	May	1.27%	2.37%	0.91%	1.91%	1.30%	1.70%	1.97%	0.55%	1.25%	1.34%	1.56%	1.95%	2.23%	2.68%	1.07%	1.03%	1.32%	2.41%	2.32%	3.55%	2.53%	1.46%	2.46%	1.46%
	Apr	1.53%	1.65%	2.35%	2.36%	2.16%	1.90%	2.61%	1.05%	2.53%	1.81%	2.63%	1.88%	2.57%	3.27%	0.91%	1.52%	2.45%	2.51%	4.07%	3.03%	3.14%	2.29%	1.81%	2.00%
	Mar	1.46%	1.65%	1.71%	0.97%	1.25%	0.81%	2.85%	0.71%	3.09%	2.07%	2.26%	1.31%	2.07%	2.12%	1.45%	1.23%	0.99%	1.87%	2.42%	3.68%	1.75%	2.13%	1.21%	1.01%
	Feb	2.72%	1.66%	2.96%	2.32%	3.39%	1.99%	2.30%	1.11%	2.00%	1.57%	2.61%	2.28%	2.70%	2.69%	1.22%	1.41%	3.83%	3.69%	3.83%	2.43%	2.94%	2.32%	3.71%	2.81%
	Jan	2.95%	4.99%	5.11%	4.12%	4.43%	3.43%	3.42%	1.67%	3.17%	3.64%	4.49%	4.10%	6.35%	5.89%	3.32%	3.74%	4.50%	4.79%	6.52%	4.49%	5.64%	4.68%	4.13%	4.03%
2020	Dec	1.55%	1.62%	1.53%	1.53%	0.79%	1.27%	2.53%	0.50%	3.38%	1.06%	1.52%	0.84%	2.81%	1.77%	0.67%	1.14%	1.16%	1.93%	2.15%	1.77%	2.12%	0.78%	1.69%	1.04%
	Nov	1.39%	1.84%	0.93%	0.85%	0.54%	0.93%	1.38%	0.59%	1.02%	0.80%	2.13%	0.97%	1.31%	1.53%	0.64%	1.04%	1.60%	1.01%	0.96%	1.20%	2.44%	0.80%	1.31%	0.54%
	Oct	1.69%	1.93%	1.88%	1.58%	1.16%	1.45%	3.17%	1.22%	0.95%	1.00%	1.98%	1.30%	3.29%	1.37%	1.04%	1.48%	1.67%	1.70%	2.50%	2.64%	2.06%	1.61%	2.09%	0.97%
	Sep	1.09%	0.94%	1.45%	1.08%	0.61%	0.70%	1.41%	0.47%	2.42%	0.76%	1.51%	0.74%	2.31%	1.51%	0.86%	0.73%	1.25%	0.91%	0.83%	1.67%	1.12%	1.46%	1.65%	0.59%
	Aug	1.50%	1.51%	0.98%	0.95%	0.36%	0.64%	1.33%	0.46%	3.14%	0.60%	1.33%	0.79%	1.28%	0.85%	0.95%	0.58%	0.73%	1.48%	1.18%	1.44%	1.73%	0.94%	1.27%	0.51%
	July	2.87%	3.48%	3.49%	3.43%	2.52%	2.62%	2.61%	1.34%	1.83%	1.97%	2.85%	3.35%	1.41%	4.45%	1.53%	3.62%	2.82%	3.37%	3.85%	5.80%	3.48%	2.72%	3.85%	2.15%
	Jun	3.65%	3.42%	3.17%	2.16%	1.68%	1.96%	1.90%	0.98%	2.76%	1.38%	3.55%	2.59%	3.33%	3.63%	1.59%	2.20%	3.20%	2.81%	4.14%	4.13%	3.69%	2.37%	3.71%	1.62%
	May	1.81%	2.27%	1.54%	1.58%	1.36%	1.15%	1.32%	0.58%	1.36%	1.79%	2.61%	1.51%	1.06%	1.79%	0.55%	1.65%	1.50%	1.61%	2.17%	2.54%	2.65%	1.60%	1.91%	1.19%
	Apr	2.35%	3.28%	2.69%	1.94%	1.53%	1.72%	1.51%	0.58%	1.18%	1.32%	1.54%	2.20%	1.37%	4.03%	0.88%	1.25%	2.48%	1.48%	2.47%	2.75%	2.24%	2.34%	3.06%	1.53%
	Mar	3.99%	7.28%	6.98%	5.05%	3.89%	4.62%	3.04%	1.98%	4.10%	3.56%	4.08%	4.83%	2.18%	8.07%	3.79%	3.47%	6.88%	2.97%	5.52%	7.30%	5.87%	4.79%	7.78%	2.82%
	Feb	2.93%	5.14%	2.76%	2.45%	2.21%	2.12%	2.74%	1.58%	4.08%	1.82%	2.17%	2.46%	1.25%	3.14%	2.66%	2.58%	2.09%	2.30%	2.64%	3.90%	2.90%	1.90%	2.43%	1.42%
	Jan	2.21%	3.44%	2.78%	1.82%	1.26%	1.32%	1.47%	0.96%	2.12%	1.88%	2.28%	2.87%	1.70%	3.53%	2.19%	1.84%	1.35%	2.33%	2.52%	2.66%	3.09%	1.95%	2.85%	1.55%

Note: **Aqua**–Aquaculture; **Build Mat**–Building Materials; **Construct**–Construction; **Cons Inv**–Construction Investment; **Dev Inv**–Development Investment; **Edu**–Education; **Tech**–Technology; **Pharma**–Pharmaceutical; **Transport**–Transportation.

**Table 6:** Raking of the monthly market risk for 24 sectors, January 2020 –September 2021.

Ranking of the market risks using VaR at a 95 per cent confidence level (1 indicates the riskiest sector, and 24 indicates the least risky sector)																									
Year	Month	Aqua	Aviation	Banking	Build Mat	Business	Construct	Cons Inv	Dev Inv	Edu	Energy	Fertilizer	Food	Mineral	Oil & Gas	Pharma	Plastic	Real Estate	Rubber	Securities	Services	Steel	Tech	Trade	Transport
2021	Sep	16	11	18	7	9	14	2	24	1	13	5	23	10	8	6	21	22	17	15	20	3	19	12	4
	Aug	8	23	6	11	10	19	15	24	22	21	5	16	13	3	17	20	9	14	1	12	4	18	7	2
	July	11	15	4	18	12	22	10	24	20	23	6	21	17	5	19	16	8	7	1	9	2	14	3	13
	Jun	6	20	5	8	7	18	19	24	4	22	9	23	11	3	21	14	16	12	1	10	2	15	17	13
	May	19	6	23	11	18	12	9	24	20	16	13	10	8	2	21	22	17	5	7	1	3	15	4	14
	Apr	21	20	12	11	14	16	6	23	8	19	5	17	7	2	24	22	10	9	1	4	3	13	18	15
	Mar	14	13	12	22	17	23	3	24	2	9	5	16	8	7	15	18	21	10	4	1	11	6	19	20
	Feb	9	20	6	14	5	19	16	24	18	21	12	17	10	11	23	22	1	4	2	13	7	15	3	8
	Jan	23	6	5	14	12	19	20	24	22	18	10	15	2	3	21	17	9	7	1	11	4	8	13	16
2020	Dec	11	10	12	13	21	15	3	24	1	18	14	20	2	8	23	17	16	6	4	7	5	22	9	19
	Nov	6	3	17	18	24	16	7	22	12	19	2	14	9	5	21	11	4	13	15	10	1	20	8	23
	Oct	11	8	9	14	20	16	2	19	24	22	7	18	1	17	21	15	12	10	4	3	6	13	5	23
	Sep	12	14	8	13	22	21	9	24	1	18	6	19	2	5	16	20	10	15	17	3	11	7	4	23
	Aug	4	3	12	14	24	19	7	23	1	20	8	17	9	16	13	21	18	5	11	6	2	15	10	22
	July	12	8	6	9	18	16	17	24	21	20	13	11	23	2	22	5	14	10	4	1	7	15	3	19
	Jun	5	8	11	17	20	18	19	24	13	23	7	14	9	6	22	16	10	12	1	2	4	15	3	21
	May	7	4	14	13	17	21	19	23	18	9	2	15	22	8	24	10	16	11	5	3	1	12	6	20
	Apr	8	2	5	12	16	13	17	24	22	20	14	11	19	1	23	21	6	18	7	4	10	9	3	15
	Mar	15	4	5	9	16	12	20	24	13	18	14	10	23	1	17	19	6	21	8	3	7	11	2	22
	Feb	5	1	7	13	16	18	8	22	2	21	17	12	24	4	9	11	19	15	10	3	6	20	14	23
	Jan	11	2	6	17	23	22	20	24	13	15	10	4	18	1	12	16	21	9	8	7	3	14	5	19
Min (riskiest)			**1**	**4**	**7**	**5**	**12**	**2**	**19**	**1**	**9**	**2**	**4**	**1**	**1**	**6**	**5**	**1**	**4**	**1**	**1**	**1**	**6**	**2**	**2**
Max (least risky)			**23**	**23**	**22**	**24**	**23**	**20**	**24**	**24**	**23**	**17**	**23**	**24**	**17**	**24**	**22**	**22**	**21**	**17**	**20**	**21**	**22**	**19**	**23**

Note: **Aqua**–Aquaculture; **Build Mat**–Building materials; **Construct**–Construction; **Cons Inv**–Construction investment; **Dev Inv**–Development investment; **Edu**–Education; **Tech**–Technology; **Pharma**–Pharmaceutical; **Transport**–Transportation.

Table 5 shows the monthly market risk among 24 sectors in Vietnam. A significant increase in the market risks among all sectors during the February-March 2020 period implies a significant response to the outbreak. However, the extent of response varies differently among sectors. The Trade sector experiences the most significant increase in the market risk by 5.35 per cent, followed by *Oil and Gas*, *Real Estate*, and *Banking* with an increase of 4.93 per cent, 4.79 per cent, and 4.22 per cent, respectively. In contrast, the market risk of *Education* increases by only 0.02 per cent, which is the lowest increase among all sectors. In summary, we note that most sectors have experienced an increase in the average market risk of about 2 per cent.

**Fig 1:**
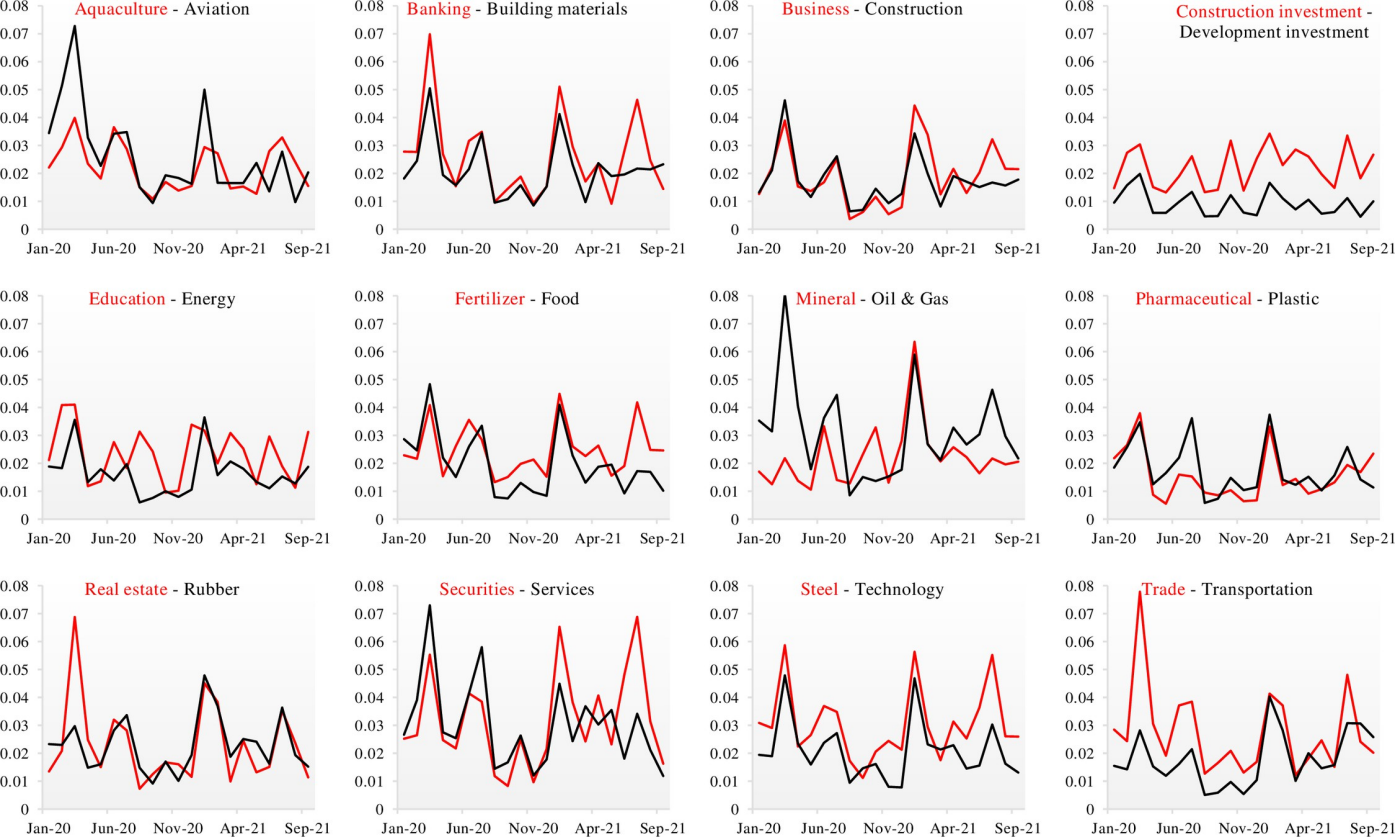
Monthly market risk for 24 sectors in Vietnam during Covid-19 pandemic. This figure presents monthly market risk for 24 Vietnamese sectors during Covid-19 pandemic in 2020 and 2021. 12 pairs of sectors are graphically illustrated. The red line presents the monthly market risks for a Vietnamese sector which appears first in the pair. The black line presents the monthly market risks for a sector which appears second in the pair.

We also illustrate the results from Table 5 in Fig 1. The market risk increases significantly in March 2020, July 2020, January 2021, and July 2021. These periods coincide with the peaks of the four Covid-19 waves in Vietnam. These findings indicate that Vietnamese sectors respond to the pandemic sensitively. *The Oil and Gas* sector has exhibited the largest risk level among all sectors during the first Covid-19 wave, while *The Services* sector exhibits a significant one during the second wave. Such high-risk levels in these two sectors may be due to the Vietnamese government’s social distancing and travel restrictions. During the last two Covid-19 waves, *The Securities* sector endures the largest potential loss. The income of the Vietnamese and households had been severely affected after the first two Covid-19 waves. In addition, a significant volume of new participants enters the stock market to trade. As a result, the risk to the sector has increased.

### 4.2. Effects of Covid-19, policy responses and macroeconomic fundamentals on market risk

Our analysis indicates a significant increase in market risk across sectors during the four Covid-19 waves from 2020 to 2021. We now examine the effects of the pandemic, policy responses to the pandemic, and macroeconomic fundamentals on market risks across sectors in Vietnam. Table 7 provides the descriptive statistics of all variables. A panel data of 24 sectors for 21 months contains from 480 to 504 observations. Market risk has a mean value of 0.022 and a standard deviation of 0.013.

**Table 7:** Descriptive statistics of variables used for analysis.

Variable	N	Mean	SD	Min	Max
*Market risk*	504	0.022	0.013	0.004	0.081
*Cases*	480	5.933	2.873	1.946	12.271
*CHI*	504	3.961	0.732	0.807	4.36
*EPU*	504	2.641	0.703	0.178	3.8
*Exchange*	504	10.049	0.007	10.033	10.064
*LIBOR*	504	0.023	0.008	0.014	0.041
*Gold*	504	7.485	0.062	7.351	7.586

Various estimation techniques, including the pooled OLS, fixed-effects, and random-effects estimators, are used in this analysis. These estimation techniques are consistent with previous studies, such as Ho et al. [16] and Li et al. [9], which examine the impacts of the Covid-19 pandemic on market risk. Table 8 presents empirical results. Various tests, such as the Sargan-Hansen and Breusch-Pagan tests, are conducted to ensure that the estimation methods are appropriate. As presented in Table 8, empirical results are relatively consistent across these estimators. However, the test statistics from the Sargan-Hansen test and Breusch-Pagan test indicate that the fixed-effects estimator is the most appropriate estimation technique for our empirical analysis.

**Table 8:** Sargan-Hansen test and Breusch and Pagan Lagrangian multiplier test.

	Pooled OLS	Fixed effects	Random effects
Variables	*Market risk*	*Market risk*	*Market risk*
*Cases*	0.00185***	0.00185***	0.00185***
	(0.000371)	(0.000371)	(0.000371)
*CHI*	-0.0311***	-0.0311***	-0.0311***
	(0.00285)	(0.00285)	(0.00285)
*EPU*	0.0236***	0.0236***	0.0236***
	(0.00194)	(0.00194)	(0.00194)
*Exchange*	-1.233***	-1.233***	-1.233***
	(0.103)	(0.103)	(0.103)
*LIBOR*	0.671***	0.671***	0.671***
	(0.0903)	(0.0903)	(0.0903)
*Gold*	-0.000861	-0.000861	-0.000861
	(0.00661)	(0.00661)	(0.00661)
Constant	12.46***	12.46***	12.46***
	(1.007)	(1.008)	(1.007)
Sargan-Hansen test			599.435*** (0.0000)
Breusch-Pagan test			210.91*** (0.0000)

Standard errors are in parentheses. ***, **, * are significant at 1, 5, and 10 per cent.

Table 9 presents our empirical results concerning these effects in two scenarios: (i) when all sectors are considered together, and (ii) each of these 24 sectors is considered. We use the fixed-effects model for the first scenario and the pooled OLS for the second scenario. Key findings are presented as follows. We report the results when all sectors are considered together from the first column of Table 9. Except for gold prices, the new Covid-19 cases, Economic Policy Uncertainty, Containment and Health, Exchange rate, and LIBOR affect the market risk. An increase in the number of new Covid-19 cases magnifies market risk. For every 1 per cent increase in the number of new Covid-19 cases over a month, the market risk for all sectors on average increases by 0.00185 per cent. This finding aligns with Li et al. [9] but is different from Ho et al. [16]. We note that Li et al. [9] use the number of new cases, whereas Ho et al. [16] use the dummy variable to proxy the pandemic. Enhancing containment and health policy mitigates market risk, whereas reducing economic policy uncertainty also reduces market risk. For every 1 per cent increase in the Containment and Health index over a month, the market risk decreases by 0.0311 per cent.

**Table 9:** The effects of the Covid-19 pandemic, policy responses and macroeconomic fundamentals on the market risks across 24 Vietnamese sectors.

**Variables**	**All sectors**	**Aqua**	**Aviation**	**Banking**	**Build Mat**	**Business**	**Construct**	**Cons Inv**	**Dev Inv**	**Edu**	**Energy**	**Fertilizer**	**Food**
*Cases*	0.00185***	0.002	0.003	0.004*	0.002	0.001	0.002	0.001	0.000	-0.001	0.000	0.002	0.001
	(0.000371)	(0.001)	(0.002)	(0.002)	(0.001)	(0.002)	(0.001)	(0.001)	(0.001)	(0.002)	(0.001)	(0.002)	(0.002)
*CHI*	-0.0311***	-0.024*	-0.059**	-0.049**	-0.036**	-0.026	-0.034**	-0.022	-0.021***	-0.022	-0.016	-0.023	-0.032*
	(0.00285)	(0.013)	(0.020)	(0.022)	(0.015)	(0.018)	(0.013)	(0.013)	(0.006)	(0.018)	(0.012)	(0.016)	(0.017)
*EPU*	0.0236***	0.017**	0.026**	0.037***	0.027***	0.027**	0.025***	0.008	0.010**	0.009	0.018**	0.018*	0.027**
	(0.00194)	(0.007)	(0.011)	(0.012)	(0.009)	(0.010)	(0.007)	(0.007)	(0.003)	(0.010)	(0.007)	(0.009)	(0.009)
*Exchange rate*	-1.233***	-0.928	-0.909	-1.325	-1.763*	-2.102*	-1.496*	-0.576	-0.707*	-1.754	-1.235	-0.725	-1.426
	(0.103)	(0.766)	(1.144)	(1.265)	(0.887)	(1.009)	(0.724)	(0.726)	(0.352)	(1.053)	(0.709)	(0.919)	(0.949)
*LIBOR*	0.671***	0.946	1.361	0.933	1.245*	1.041	1.007*	-0.230	0.399	0.848	0.359	0.017	0.960
	(0.0903)	(0.546)	(0.816)	(0.902)	(0.632)	(0.720)	(0.516)	(0.518)	(0.251)	(0.751)	(0.505)	(0.656)	(0.677)
*Gold*	-0.000861	0.019	0.020	0.000	0.045	0.004	0.030	-0.035	0.015	0.009	-0.040	-0.040	0.014
	(0.00661)	(0.055)	(0.083)	(0.091)	(0.064)	(0.073)	(0.052)	(0.053)	(0.025)	(0.076)	(0.051)	(0.066)	(0.069)
Constant	12.46***	9.224	9.131	13.397	17.431*	21.118*	14.865*	6.143	7.055*	17.639	12.731*	7.636	14.271
	(1.007)	(7.505)	(11.220)	(12.404)	(8.693)	(9.894)	(7.096)	(7.120)	(3.450)	(10.319)	(6.947)	(9.014)	(9.308)
N	480	20	20	20	20	20	20	20	20	20	20	20	20
R-squared	0.377	0.545	0.707	0.613	0.587	0.531	0.655	0.413	0.650	0.376	0.544	0.439	0.582
**Variables**	**All sectors**	**Mineral**	**Oil & Gas**	**Pharma**	**Plastic**	**Real Estate**	**Rubber**	**Securities**	**Services**	**Steel**	**Tech**	**Trade**	**Transport**
*Cases*	0.00185***	-0.002	0.005*	0.001	0.002	0.003*	0.000	0.004	0.003	0.005**	0.001	0.005**	0.001
	(0.000371)	(0.002)	(0.002)	(0.001)	(0.002)	(0.002)	(0.002)	(0.003)	(0.002)	(0.002)	(0.002)	(0.002)	(0.001)
*CHI*	-0.0311***	-0.002	-0.040	-0.034**	-0.036**	-0.047**	-0.020	-0.031	-0.045*	-0.044*	-0.020	-0.052**	-0.012
	(0.00285)	(0.021)	(0.025)	(0.013)	(0.017)	(0.018)	(0.020)	(0.032)	(0.024)	(0.021)	(0.017)	(0.019)	(0.015)
*EPU*	0.0236***	0.020	0.034**	0.018**	0.015	0.043***	0.020*	0.027	0.028*	0.024*	0.026**	0.043***	0.019**
	(0.00194)	(0.012)	(0.014)	(0.007)	(0.009)	(0.010)	(0.011)	(0.018)	(0.013)	(0.012)	(0.010)	(0.011)	(0.008)
*Exchange*	-1.233***	-2.141*	-0.802	-1.533*	-0.584	-1.809	-1.526	-0.803	-0.681	-0.522	-1.101	-1.477	-1.680*
	(0.103)	(1.202)	(1.452)	(0.733)	(0.955)	(1.035)	(1.135)	(1.849)	(1.365)	(1.224)	(1.005)	(1.102)	(0.874)
*LIBOR*	0.671***	0.141	0.645	0.969*	0.522	1.034	0.533	0.046	0.397	0.383	0.398	1.408*	0.743
	(0.0903)	(0.857)	(1.036)	(0.523)	(0.681)	(0.738)	(0.810)	(1.319)	(0.973)	(0.873)	(0.717)	(0.786)	(0.623)
*Gold*	-0.000861	0.030	-0.061	0.020	0.020	0.011	0.021	-0.061	-0.048	-0.003	-0.030	0.047	-0.006
	(0.00661)	(0.087)	(0.105)	(0.053)	(0.069)	(0.075)	(0.082)	(0.134)	(0.099)	(0.088)	(0.073)	(0.080)	(0.063)
Constant	12.46***	21.281*	8.573	15.335*	5.814	18.152*	15.213	8.584	7.315	5.375	11.302	14.552	16.914*
	(1.007)	(11.784)	(14.238)	(7.182)	(9.364)	(10.149)	(11.132)	(18.126)	(13.380)	(11.997)	(9.856)	(10.801)	(8.569)
N	480	20	20	20	20	20	20	20	20	20	20	20	20
R-squared	0.377	0.387	0.589	0.609	0.412	0.718	0.255	0.341	0.560	0.494	0.508	0.721	0.543

Note: **Aqua**–Aquaculture; **Build Mat**–Building Materials; **Construct**–Construction; **Cons Inv**–Construction Investment; **Dev Inv**–Development Investment; **Edu**–Education; **Tech**–Technology; **Pharma**–Pharmaceutical; **Transport**–Transportation. Standard errors in parentheses; ***, **, * are significant at 1, 5, and 10 per cent.

Meanwhile, the market risk drops by 0.0236 per cent for every 1 per cent fall in the EPU index over a month. These findings are also consistent with previous studies [25, 28, 29] in which market volatility reduces with improved containment and health policy and decreased economic policy uncertainty during the period. For macroeconomic fundamentals, an increase in the exchange rate (USD/VND), representing a depreciation of the local currency VND against the $ US, is associated with a reduction in market risk. However, we also find that an increased interest rate heightens market risk. For example, when the local currency VND depreciates 1 per cent against the $ US, the market risk will decrease by 1.233 per cent. Also, a one per cent increase in interest rate, proxied by LIBOR, increases the market risk by 0.671 per cent. These findings also align with previous studies [31, 33, 34].

We now report and discuss the empirical results when each of these 24 sectors is considered. The effects on the market risk appear to be as consistent as all sectors are considered together. The market risks from *Building Materials*, *Construction*, *Pharmaceutical*, and *Trade* sectors exhibit the same effects when all sectors are considered together. The pandemic affects the *Oil and Gas*, *Steel*, and *Trade* sectors the most, followed by *Banking* and *Real Estate* sectors. The policy responses to the pandemic affect many sectors in Vietnam. In particular, the containment and health policy significantly reduces the market risk in 13 out of 24 sectors. A reduction in the EPU index reduces market risks across 18 out of 24 sectors. Exchange rates also affect the market risk for 7 out of 24 sectors in Vietnam. Gold price does not appear to affect the market risk from any sector in Vietnam.

## 5. Concluding remarks and implications

Vietnam has undergone four waves of the Covid-19 pandemic in the past 24 months, which has dramatically disturbed the risks of various sectors in Vietnam. While empirical studies on the effects of the pandemic have been extensively conducted, the focus on the market risk across sectors has largely been ignored in the current literature, especially in Vietnam. As such, this study examines the effects of the pandemic, policy responses and macroeconomic fundamentals on the market risk among sectors in Vietnam from 2012 to 2021. We employ the VaR and CVaR measures to estimate the market risks for 24 sectors. We first measure the yearly market risk from 2012 to 2021 for each sector in Vietnam to examine how significant the market risks have changed in the past decade for these sectors. We then examine the effects of the pandemic, policy responses, and macroeconomic fundamentals on the market risks.

Our results indicate that *Aviation* is the riskiest sector, whereas *Pharmaceutical* shows the lowest level of market risk during the 2012–2021 period. Furthermore, the rankings of sectoral market risks have been significantly volatile between 2012 and 2021. *Pharmaceutical* is the only sector demonstrating the rankings’ stability during the research period. Our results confirm a significant response from all sectors to the Covid-19 outbreaks in Vietnam. Specifically, the market risk increases significantly during the peaks of the four Covid-19 waves in Vietnam. *The Oil and Gas* sector shows the largest market risk level during the first Covid-19 wave, while *The Service*s sector suffers the most significant potential loss during the second wave. During the last two Covid-19 waves in 2021, *The Securities* sector suffered the most significant potential loss.

Our results indicate that the Vietnamese sectors show a different market risk. Company executives and practitioners should consider this valuable information when making decisions. Companies in the riskiest sectors such as *Aviation*, *Oil and Gas*, and *Services* should focus on their risk management strategies, including product diversification. For example, airlines may need to design an appropriate hybrid model to diversify the revenue streams from passengers and cargo, particularly during an extreme event such as the Covid-19 pandemic. In addition to improving reserves, oil and gas companies should invest in refining and petrochemicals that concentrate on deep processing and the quality of petroleum products. Service companies should leverage online platforms instead of in-person operations disrupted by the pandemic. For example, digital tourism can become an alternative to traditional tourism. In addition, lower-risk sectors such as *Pharmaceutical* will also need to initiate and implement business strategies to deal with the worst possible scenarios from market risks in the future.

Our empirical analysis confirms a significant relationship between the sectoral market risk and the new Covid-19 cases, economic policy uncertainty, containment and health, exchange rate, and interest rate (LIBOR). We find that increasing the number of new Covid-19 cases increases sectoral market risk in Vietnam. Decreased market risks are associated with improved economic policy uncertainty and improved containment and health policy. Our results also indicate that market risks across sectors in Vietnam increase when the Vietnam Dong (VND) appreciates against the $ US. In addition, an increased interest rate leads to increased sectoral market risks. In conclusion, the Covid-19 pandemic affects the market risks for all sectors in Vietnam. *Oil and Gas*, *Steel*, and *Trade*, followed by *Banking* and *Real estate*, are the most affected sectors. These findings provide important policy implications for the Vietnamese government, policymakers, and investors.

The Vietnamese government should provide the necessary financial and non-financial support to affected sectors to help them recover from the pandemic. Stabilizing domestic gasoline prices should be implemented as gasoline is a significant cost component of the affected sectors. Maintaining this stability will support the performance of these sectors and the entire market. Domestic trade and consumption should be promoted to support the sectors. The government should continue supporting small-medium enterprises in joining industrial clusters and value chains and creating value. For example, the government can implement an interest rate support package for enterprises facing difficulties due to the Covid-19 pandemic. The support package is particularly of help for those that are genuinely struggling but have the potential to recover and grow. Innovative entrepreneurship should also be promoted to create breakthrough business models and market approaches, thereby overcoming the limitations of old models in the context of escalated market risk from the Covid-19 crisis.

The government should implement containment and health policies more assertively. Clear plans for several scenarios should be implemented to minimize uncertainty, decreasing market risk for the entire market and sectors. In addition, the State Bank of Vietnam should flexibly manage the exchange rate to effectively affect the foreign exchange market, such as stabilizing exchange rates and enhancing liquidity while still ensuring the international competitive advantage. Investors should know the high-risk sectors for appropriate portfolio diversification strategies, thereby delivering expected returns. Risk-averse investors should adopt hedging strategies to protect their portfolios against significant market risk. Investment decisions in the stock market should consider exchange rates and interest rates.

Our study has potential limitations that offer possible directions for further research. First, market risk can be estimated using measures such as expected shortfall and others. Using various measures for market risk provides robustness to the estimates. Second, a nonlinear relationship between market risk and the pandemic, policy responses and macroeconomic fundamentals may also exist. A mechanism for this nonlinear relationship can be initiated and tested. Third, previous studies have examined the connectedness among several markets with the exposure to market risk. However, the connectedness of market risk across sectors remains unaddressed. These limitations and gaps should be thoroughly examined in future studies.

## Supporting information

S1 Dataset (RAR)
