# Supplementary material for: The impacts of the Covid-19 pandemic, policy responses and macroeconomic fundamentals on market risks across sectors in Vietnam
Source: PLoS One. 2022 Aug 23;17(8):e0272631. doi: 10.1371/journal.pone.0272631 (PMC9397878; doi:10.1371/journal.pone.0272631)
Supplement: Attachment — Submitted filename: PONE-D-22-08126.docx [file pone.0272631.s002.docx]

Overall Comments

The theme of the paper is very interesting and has a lot of practical implications. However, following are some suggestions to improve the paper

1. The study offers an original idea and novel contribution to the existing field of research. There are research gaps explicitly stated but the rationale of study got to be established.
2. The authors should explain that the choice of variables and macroeconomic indicators in detail. What is economic justification of analyzing relationship between sectoral risk, COVID-19 pandemic effect, policy response and macroeconomic indicators in Vietnam. Explain
3. Please enrich the literature with some recent studies other than research conducted in context of Vietnam for wider readership. There are many studies conducted in given context at international level. How your study is different from existing literature? Explain. See and cite recent research work on COVID-19 pandemic and economy by Qureshi. F (2021).
4. Are the empirical findings similar or different to the findings by earlier studies in the current research framework? Explain
5. The conclusion section should be rewritten. Provide clear and precise information in the conclusion section with focus on policy implications

References:

Qureshi, F. (2021). COVID-19 pandemic, economic indicators and sectoral returns: evidence from US and China. *Economic Research-Ekonomska Istraživanja*, 1-31.
