# Supplementary material for: The impacts of the Covid-19 pandemic, policy responses and macroeconomic fundamentals on market risks across sectors in Vietnam
Source: PLoS One. 2022 Aug 23;17(8):e0272631. doi: 10.1371/journal.pone.0272631 (PMC9397878; doi:10.1371/journal.pone.0272631)
Supplement: Attachment — Submitted filename: Response to Reviewers.docx [file pone.0272631.s003.docx]

**A Summary of Responses to Reviewers’ Comments**

**“The impacts of Covid-19 pandemic, policy responses and macroeconomic fundamentals on market risks across sectors in Vietnam”**

Dear Editor-in-Chief, the Academic Editor and the Reviewers,

Thank you very much for the opportunity to revise and resubmit our paper. We consider that all comments from you are very constructive and insightful. As such, the response to each of the comments is incorporated into the revised version of the paper. Our corrections and additional discussions in response to your comments are highlighted in yellow for your convenience.

Thank you very much and all the very best.

**Academic Editor:**

**Comment 1**:	*Please ensure that your manuscript meets PLOS ONE's style requirements, including those for file naming. The PLOS ONE style templates can be found at* 
[*https://journals.plos.org/plosone/s/file?id=wjVg/PLOSOne_formatting_sample_main_body.pdf*](https://journals.plos.org/plosone/s/file?id=wjVg/PLOSOne_formatting_sample_main_body.pdf) *and* 
[*https://journals.plos.org/plosone/s/file?id=ba62/PLOSOne_formatting_sample_title_authors_affiliations.pdf*](https://journals.plos.org/plosone/s/file?id=ba62/PLOSOne_formatting_sample_title_authors_affiliations.pdf)

***Response***:	Thank you very much for your comment. Please be advised that we have reformatted our manuscript to meet PLOS ONE’s style requirements.

**Comment 2**:	*We note that the grant information you provided in the ‘Funding Information’ and ‘Financial Disclosure’ sections do not match. When you resubmit, please ensure that you provide the correct grant numbers for the awards you received for your study in the ‘Funding Information’ section.*

***Response***:	Thank you very much for this guidance. We will ensure that the grant information is consistent.

**Comment 3**:	*We note that you have indicated that data from this study are available upon request. PLOS only allows data to be available upon request if there are legal or ethical restrictions on sharing data publicly. For more information on unacceptable data access restrictions, please see*

[*http://journals.plos.org/plosone/s/data-availability#loc-unacceptable-data-access-restrictions*](http://journals.plos.org/plosone/s/data-availability#loc-unacceptable-data-access-restrictions)*.*

***Response***:	Thank you for your comment. We have now made the data available with the submission of this revised paper.

**Comment 4**:	*In your revised cover letter, please address the following prompts:*

*a) If there are ethical or legal restrictions on sharing a de-identified data set, please explain them in detail (e.g., data contain potentially sensitive information, data are owned by a third-party organization, etc.) and who has imposed them (e.g., an ethics committee). Please also provide contact information for a data access committee, ethics committee, or other institutional body to which data requests may be sent.*

*b) If there are no restrictions, please upload the minimal anonymized data set necessary to replicate your study findings as either Supporting Information files or to a stable, public repository and provide us with the relevant URLs, DOIs, or accession numbers. For a list of acceptable repositories, please see*

[*http://journals.plos.org/plosone/s/data-availability#loc-recommended-repositories*](http://journals.plos.org/plosone/s/data-availability#loc-recommended-repositories)*. We will update your Data Availability statement on your behalf to reflect the information you provide.*

***Response***:	Thank you for your comment. We have now made the data available with the submission of this revised paper.

**Reviewer 1:**

**Comment 1**:	*This is a very well written paper in terms of language, robust measures of econometrics analysis is properly executed. Results have also been analyzed critically and reflect policy implication.* *Therefore, I recommend minor revision as follows:*

***Response***:	Thank you very much for your encouragement.

**Comment 2**:	*The length of the paper should be reduced.*

***Response***:	Thank you very much for your comment. We have now reduced the length of the paper.

**Comment 3**:	*Stress on the most important contribution of your study.*

***Response***:	Thank you very much for your suggestion. Please be advised that we have now redrafted and emphasized on the most important contributions of our study in the Introduction section on pages 2 – 4 of the revised paper.

**Comment 4**:	*Update the recent literature on Covid-19.*

***Response***:	Thank you very much for your comment and the recent studies related to the Covid-19 pandemic. Please be advised that we have now discussed and included these suggested studies to our paper.

**Reviewer 2:**

**Comment 1**:	*The theme of the paper is very interesting and has a lot of practical implications. However, following are some suggestions to improve the paper*

***Response***:	Thank you very much for your encouragement. We have now incorporated responses to your constructive comments in our revised paper.

**Comment 2**:	*The study offers an original idea and novel contribution to the existing field of research. There are research gaps explicitly stated but the rationale of study got to be established.*

***Response***:	Thank you very much for your comment. We have redrafted and established the rationale of our study in the Introduction section on pages 2-4 of the revised paper.

**Comment 3**:	*The authors should explain that the choice of variables and macroeconomic indicators in detail. What is economic justification of analyzing relationship between sectoral market risk, COVID-19 pandemic effect, policy response and macroeconomic indicators in Vietnam. Explain*

***Response***:	Thank you very much for your insightful comment. Please be advised that we have now explained the choice of variables and macroeconomic fundamentals which are in line with previous studies on pages 6 – 7 of the revised paper.

**Comment 4**:	*Please enrich the literature with some recent studies other than research conducted in context of Vietnam for wider readership. There are many studies conducted in given context at international level. How your study is different from existing literature? Explain. See and cite recent research work on COVID-19 pandemic and economy by Qureshi. F (2021).*

***Response***:	Thank you very much for your insightful comment. We have added the recent study by Qureshi. F (2021), as well as recent studies suggested by Reviewer #1. We have also discussed additional studies on the relationship between the Covid-19 pandemic, policy responses, macroeconomic fundamentals and stock returns and volatility.

**Comment 5**:	*Are the empirical findings similar or different to the findings by earlier studies in the current research framework? Explain*

***Response***:	Thank you very much for your insightful comment. The focus of our paper is on the market risk across sectors in Vietnam. Our empirical findings are in line with Li et al. [10] but different from Ho et al. [16]. We note that Li et al. [10] also use the number of new cases in their analysis whereas Ho et al. [16] use dummy variables for the Covid-19 months to proxy for the Covid-19 pandemic. Our findings are also consistent with previous studies [25, 28, 29] in which market volatility is lower with improved containment and health policy and economic policy uncertainty during the Covid-19 period. In addition, our findings are also consistent with those from previous studies [31, 33, 34] which found significant relationship between exchange rate, LIBOR and stock market returns. These discussions are now incorporated in the revised paper.

**Comment 6**:	*The conclusion section should be rewritten. Provide clear and precise information in the conclusion section with focus on policy implications*

***Response***:	Thank you very much for your insightful comment. Please be advised that we have redrafted the Conclusion section with a focus on the policy implications for the Vietnamese government, policymakers, and investors on pages 28-30 of the revised paper.

**Reviewer 3:**

**Comment 1**:	*Please present the descriptive statistics for all variables.*

***Response***:	Thank you very much for your suggestion. Please be advised that we have now added the descriptive statistics for all variables in Table 7.

**Comment 2**:	*There is no EPU data for Vietnam (see* [*https://www.policyuncertainty.com/*](https://www.policyuncertainty.com/)*). Did you use GLOBAL EPU?*

***Response***:	Thank you very much for your comment. Yes, we used the global EPU.

**Comment 3**:	*It is not clear which method you used to obtain the findings in Table 7. Please also present the test statistics of the assumptions of the method you used.*

***Response***:	We use fixed-effects estimation for the first scenario when all Vietnamese sectors are considered. The pooled OLS estimation is then used in the second scenario when each sector is considered separately. We have also presented the test statistics to support the preferences among pooled OLS, fixed-effects, and random-effects estimators in Table 8.

**Comment 4**:	*The author(s) should discuss possible directions for further research.*

***Response***:	Thank you for your insightful comment. Please be advised that we have discussed possible directions for further research on page 30 of this revised paper.
