# Supplementary material for: The impacts of the Covid-19 pandemic, policy responses and macroeconomic fundamentals on market risks across sectors in Vietnam
Source: PLoS One. 2022 Aug 23;17(8):e0272631. doi: 10.1371/journal.pone.0272631 (PMC9397878; doi:10.1371/journal.pone.0272631)
Supplement: Attachment — Submitted filename: Response to Reviewers R&R2.docx [file pone.0272631.s004.docx]

**A Summary of Responses to Reviewers’ Comments**

**“The impacts of Covid-19 pandemic, policy responses and macroeconomic fundamentals on market risks across sectors in Vietnam”**

Dear Editor-in-Chief, the Academic Editor and the Reviewers,

Thank you very much for the opportunity to revise and resubmit our paper in Round 2. We are very happy to learn that the other five reviewers are satisfied with our revision in Round 1. Nevertheless, comments from Reviewer # 4 in this second-round review are very constructive and insightful. The responses to each of the comments are incorporated into the revised paper.

We consider that all comments are very constructive and insightful. Therefore, the response to each comment is incorporated into the revised version of the paper. Our corrections and additional discussions in response to your comments are highlighted in yellow for your convenience.

Thank you very much, and all the very best.

**Reviewer 2:**

**Comment 1**:	*No comments. I am satisfied with the comments addressed.*

***Response***:	Thank you very much for your encouragement.

**Reviewer 4:**

**Comment 1**:	*The main purpose of this study is to analyse market risks of Vietnamese sectors and determine the driving factors of market risk. I enjoyed reading the paper and found the results quite interesting. I suppose the paper has been reviewed before, and it seems the authors have carefully addressed the reviewers' comments. I have two minor suggestions/comments though*

***Response***:	Thank you very much for your comment and encouragement. The paper has undergone the first-round review, and we have thoroughly addressed the reviewers’ comments in Round 1.

**Comment 2**:	*The paper has potential implications and insights for investors and policymakers alike. However, as it stands, it lacks these implications. The authors could expand more on what their results offer for different stakeholders.*

***Response***:	Thank you very much for your insightful comment and suggestion. Please be advised that we have discussed the implications for the government and investors on pages 25-26. We have also added the implications for different stakeholders, as suggested on page 27 of the revised paper.

**Comment 3**:	*How do the authors make sure their regression models are well-specified? I suggest the authors conduct some diagnostics tests and verify their regression results are reliable.*

***Response***:	Thank you very much for your excellent question and suggestion. We specify our regression model based on previous studies which confirm the significant effects of the Covid-19 pandemic [22-25], policy responses [23-30] and macroeconomic fundamentals [31-34] on stock market return and volatility. We follow Ho et al. [16] and Li et al. [10] using different estimation techniques, including the pooled OLS, fixed-effects, and random-effects estimators. We have used the test statistics from the Sargan-Hansen and Breusch-Pagan tests to identify the most appropriate for our empirical analysis. On balance, we consider that our model is well specified, and the estimation techniques are appropriate. The discussions are on page 22 of the revised manuscript.

Please be advised that all the above references are from the reference list of the revised paper. We have reproduced these references at the end of this document for your convenience.

**Comment 4**:	*Finally, what do the results from this study add to our understanding? The authors should compare their findings with previous studies to position their work in the existing literature.*

***Response***:	Thank you very much for your excellent question and comment. Our results provide a comprehensive view of the changes in market risk across Vietnamese sectors over the last decade, including the Covid-19 period. The results also signify the significant effects of the Covid-19 pandemic, policy responses and macroeconomic fundamentals on the market risk across sectors in Vietnam. Our empirical findings are in line with Li et al. [10] but different from Ho et al. [16]. However, our findings are also consistent with previous studies [25, 28, 29], in which market volatility is lower with improved containment and health policy and economic policy uncertainty during the Covid-19 period. In addition, our findings are also consistent with those from previous studies [31, 33, 34], which found a significant relationship between exchange rate, LIBOR and stock market returns. These discussions are presented on pages 23-24 of the revised paper.

**Reviewer 5:**

**Comment 1**:	*Authors have addressed all comments from the first-round review. However, authors can strengthen their contribution section when proofreading the article for publication.*

***Response***:	Thank you very much for your comment. Please be advised that we have carefully proofread the entire paper, particularly strengthening the contributions of the paper on page 3 of the revised paper.

**Reviewer 6:**

**Comment 1**:	*The authors have addressed all the necessary comments for the earlier version of the manuscript. The readability of the paper now is much better.*

***Response***:	Thank you very much for your encouragement.

**References**

10.	Li MC, Lai CC, Xiao L. Did COVID-19 increase equity market risk exposure? Evidence from China, the UK, and the US. Applied economics letters. 2022;29(6):567-71.

16.	Ho CM, Pham TT, Nguyen HL-P, Vo DH. Does the COVID-19 pandemic matter for market risks across sectors in Vietnam? Heliyon. 2021;7(12):e08453-e.

22.	Al-Awadhi AM, Alsaifi K, Al-Awadhi A, Alhammadi S. Death and contagious infectious diseases: Impact of the COVID-19 virus on stock market returns. Journal of Behavioral and Experimental Finance. 2020;27:100326.

23.	Ashraf BN. The economic impact of government interventions during the COVID-19 pandemic: International evidence from financial markets. Journal of Behavioral and Experimental Finance. 2020;27:100371.

24.	Guven M, Cetinguc B, Guloglu B, Calisir F. The effects of daily growth in COVID-19 deaths, cases, and governments’ response policies on stock markets of emerging economies. Research in international business and finance. 2022;61:101659-.

25.	Ibrahim I, Kamaludin K, Sundarasen S. COVID-19, Government Response, and Market Volatility: Evidence from the Asia-Pacific Developed and Developing Markets. Economies. 2020;8(4).

26.	Batabyal S, Killins R. Economic policy uncertainty and stock market returns: Evidence from Canada. Journal of economic asymmetries. 2021;24:e00215.

27.	Chang C-P, Feng G-F, Zheng M. Government Fighting Pandemic, Stock Market Return, and COVID-19 Virus Outbreak. Emerging markets finance & trade. 2021;57(8):2389-406.

28.	Kundu S, Paul A. Effect of economic policy uncertainty on stock market return and volatility under heterogeneous market characteristics. International review of economics & finance. 2022;80:597-612.

29.	Sharif A, Aloui C, Yarovaya L. COVID-19 pandemic, oil prices, stock market, geopolitical risk and policy uncertainty nexus in the US economy: Fresh evidence from the wavelet-based approach. International review of financial analysis. 2020;70:101496.

30.	Xu Y, Wang J, Chen Z, Liang C. Economic policy uncertainty and stock market returns: New evidence. The North American journal of economics and finance. 2021;58:101525.

31.	Beckmann J, Czudaj RL. Exchange rate expectation, abnormal returns, and the COVID-19 pandemic. Journal of economic behaviour & organisation. 2022;196:1-25.

32.	Drake PP. The gold-stock market relationship during COVID-19. Finance research letters. 2022;44:102111-.

33.	Lim SY, Sek SK. Exploring the Inter-relationship between the Volatilities of Exchange Rate and Stock Return. Procedia economics and finance. 2014;14:367-76.

34.	Rizwan MF, Khan SU. Stock Return Volatility in Emerging Equity Market (Kse): The Relative Effects of Country and Global Factors. International Review of Business Research Papers. 2007;3(2):362-75.
